# Supplementary material for: Toward a human brain extracellular vesicle atlas: Characteristics of extracellular vesicles from different brain regions, including small RNA and protein profiles
Source: Interdiscip Med. 2023 Aug 15;1(4):e20230016. doi: 10.1002/INMD.20230016 (PMC10712435; doi:10.1002/INMD.20230016)
Supplement: Supplementary file 1 — Supporting Information S1 [file INMD-1-e20230016-s001.docx]

Figure S1 Western blots of Alix, CD63, CD9, and calreticulin associated with brain homogenate after collagenase digestion (BHC), differential centrifugation pellets, size exclusion chromatography (SEC) bdEVs, and protein (P) fractions. WBs are representative of three independent human tissue EV separations from additional samples obtained from the brain bank for the purpose of protocol reproducibility assessment.

Figure S2 RNA biotypes of bdEVs from different regions. Percent of mapped reads for the RNA biotypes abundant across the regions (a), underrepresented across the regions (b), and other identified RNA biotypes in bdEVs (c).
