# Supplementary figures and images for: Toward a human brain extracellular vesicle atlas: Characteristics of extracellular vesicles from different brain regions, including small RNA and protein profiles
Source: Interdiscip Med. 2023 Aug 15;1(4):e20230016. doi: 10.1002/INMD.20230016 (PMC10712435; doi:10.1002/INMD.20230016)

Figure S1

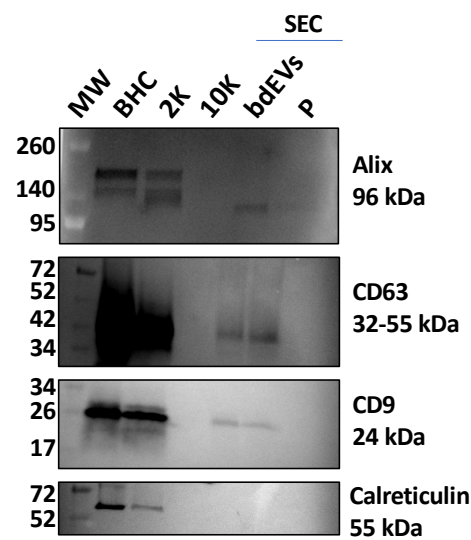

Figure S2

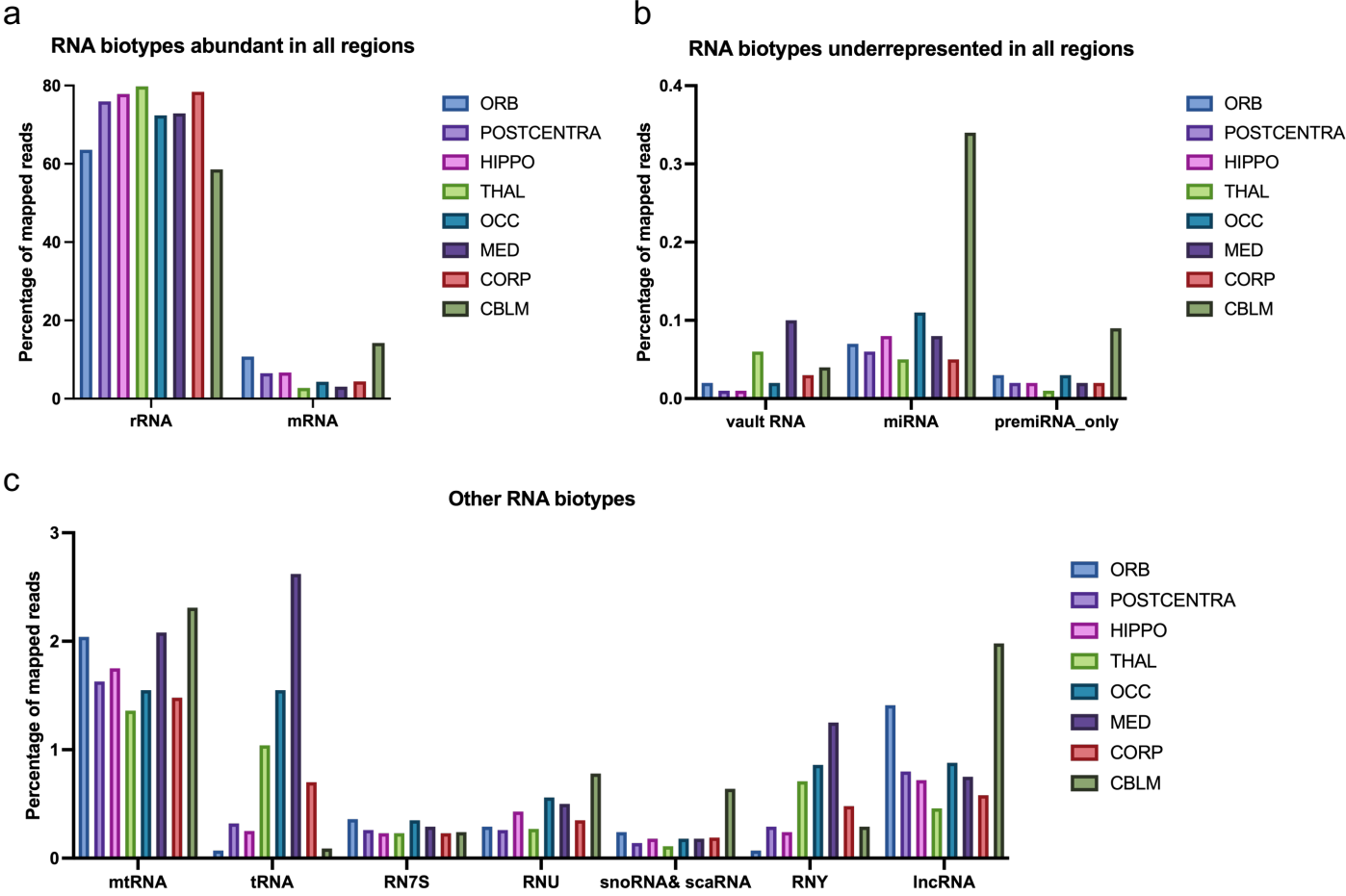

Supplement: Supplementary file 2 — Supporting Information S2 [file INMD-1-e20230016-s002.pdf]
